# Relationship between sense of coherence and subjective well-being among family caregivers of breast cancer patients: a latent profile analysis

**DOI:** 10.3389/fpsyt.2024.1515570

**Published:** 2025-01-21

**Authors:** Hui Wang, Yuxia Wu, Xuefang Huang, Haiou Yan

**Affiliations:** ^1^ Department of Oncology, Affiliated Hospital of Nantong University, Nantong, Jiangsu, China; ^2^ Department of Interventional Oncology, Affiliated Hospital of Nantong University, Nantong, Jiangsu, China; ^3^ Department of Nursing, Affiliated Hospital of Nantong University, Nantong, Jiangsu, China

**Keywords:** breast cancer, cancer caregiver, sense of coherence, subjective well-being, latent profile analysis

## Abstract

**Objective:**

Sense of coherence (SOC) assists cancer-affected caregivers in overcoming challenges in the process of caregiving and may potentially influence an individual’s subjective well-being (SWB). This study aimed to explore distinct SOC profiles among caregivers of breast cancer patients, identify the distribution differences of these profiles in sociodemographic and clinical characteristics, and explore their relationship with SWB.

**Methods:**

A total of 360 patients with caregivers of breast cancer patients from one tertiary hospitals in Jiangsu completed the Sociodemographic and clinical characteristics, the Sense of Coherence Scale (SOC-13), and the General Subjective Well-Being Schedule (GWB). Mplus 8.3 for latent profile was performed to identify SOC classes. Multivariate logistic regression was used to analyze the impact of various factors on the different categories, and ANOVA was applied to compare the SWB among caregivers of different categories.

**Results:**

Three latent profiles of SOC were identified: the “low sense of coherence-meaning group” (7.9%), the “moderate sense of coherence-manageability group” (37.3%), and the “high sense of coherence-optimism group” (54.7%). Age, residence, health status, financial pressure, caregiving duration, and breast cancer stage significantly influenced the distribution of SOC in caregivers of breast cancer patients. The SWB level differed significantly among these three categories.

**Conclusion:**

This study identified three distinct classes of SOC among caregivers. It is recommended that health care providers screen caregivers with diverse profiles of SOC and pay more attention to young, rural, long-term caregiving duration, heavy economic burden, and caregivers in poor physical condition.

## Introduction

Throughout the world, breast cancer is a prevalent form of cancer that affects women and has a significant effect on their quality of life (1). According to the 2020 Global Cancer Statistics, China had 416,000 new cases of breast cancer and 117,000 deaths, exceeding lung cancer to become the most common cancer (11.7%) with a mortality rate ranking in the top five (6.9%) worldwide (2). While the rapid advancement of diagnostic and treatment technologies, has improved breast cancer patients’ five-year relative survival rate of around 73%, and family caregivers are the primary caregivers during the recovery period (3). Cancer is considered “our disease” that has a significant impact on the families of patients (4). Long-term and comprehensive care from caregivers is necessary during the entire process of diagnosing, treating, and recovering breast cancer patients (5). When the caregiver’s capacity exceeds the demands of caregiving tasks, it will create a burden of care that will affect their physical and mental health, quality of life, and further affecting subjective well-being. However, research on the mental health issues of family caregivers for long-term survivors is still limited. Subjective well-being (SWB) refers to an individual’s positive attitude and feeling towards their current living situation based on their own standards, which encompasses life satisfaction and happiness. It is an important indicator for measuring quality of life and mental health (6). A high level of SWB can not only enhance an individual’s quality of life, but also prevent diseases and lower mortality rates (7). Evidently, taking on the role of a caregiver can significantly reduce SWB, and the greater the caregiving stress, the more obvious the decline in SWB (8). Addressing the long-term mental health issues of caregivers is crucial for healthcare professionals and researchers.

With the development of positive psychology, scholars have gradually begun to explore factors that promote the physical and mental health of caregivers. It was discovered by the study that a sense of coherence is closely linked to positive psychological factors like caregivers’ mental health, well-being, and post-traumatic growth (9). Antonovsky (10) proposed the concept of sense of coherence (SOC) in 1979, which refers to an individual’s confident feeling of maintaining control and meaning in the face of stress. SOC encompasses understanding life’s stress and pressure, utilizing internal and external resources, and recognizing the meaning of life. It is a stable internal psychological tendency and a psychological protection mechanism for individuals. Caregivers with a high level of SOC can flexibly adopt appropriate strategies to adapt to specific environmental demands, thereby alleviating negative emotions such as anxiety and depression caused by caregiving, improving their quality of life, and promoting the recovery of patients (11). Therefore, the physiological and psychological pressure of caregivers can be effectively relieved by enhancing SOC. Current oncology research has mainly examined the SOC of caregivers for patients with gastric cancer (12) and lung cancer (13). Limited studies on spouses of breast cancer patients have shown that SOC is influenced by factors such as personality traits, perceived social support, and caregiver burden (14). According to the salutogenic theory (10), SOC possesses unique characteristics that need to be categorized based on their functions, rather than viewed as a single group. This theory emphasizes the role of protective psychological traits in minimizing the detrimental impact of caregivers on the caregiving process and enhancing overall well-being.

However, the current research exploring the SOC among caregivers is limited. Previous studies mainly measured the level of SOC in caregivers using the total score of a scale, which cannot accurately reflect the differences among groups with different levels of SOC, thereby affecting the effectiveness of interventions. As interest in personalized care continues to grow, tailored interventions based on specific group characteristics are becoming increasingly popular (4). Therefore, studying the categories of SOC among caregivers for breast cancer patients can help healthcare professionals accurately identify those with lower levels of SOC and intervene in groups. Latent Profile Analysis (LPA) is an individual-centered research approach that emphasizes heterogeneity within samples (15). It identifies subgroups within a population based on individuals’ responses to each item and uses rigorous fit indices to evaluate the latent profile model, ensuring maximum heterogeneity between groups and minimum heterogeneity within groups (16). This improves the accuracy and objectivity of grouping and helps to more intuitively and clearly demonstrate group differences, thereby providing personalized healthcare services to caregivers. Given the previous research gap in neglecting SOC patterns and related factors, this study utilizes LPA to identify different characteristics of SOC among caregivers of breast cancer patients. The study aims to reveal the sociodemographic and clinical characteristics related to these features, and determine the impact of SOC in each potential category on SWB, the findings will provide strategies to improve the psychological health of caregivers.

## Methods

### Study design, setting, and participants

This quantitative cross-sectional exploratory study was conducted at a tertiary hospital in Jiangsu Province, China. During the time period from November 2023 to April 2024, 360 family caregivers of breast cancer patients were invited to the study.

The inclusion criteria were as follows: (1) caregivers of patients diagnosed with breast cancer based on pathological sections or cytology; (2) age ≥ 18 years; (3) caregiving duration exceeding 1 month; (4) patient’s spouse, children, parents, or other direct relatives; (5) responsible for the primary caregiving tasks for the patient; (6) possess normal communication abilities, and both the caregiver and patient consent to participate in this study. Exclusion criteria: (1) history of mental illness or severe cognitive impairments; (2) recent occurrence of other severe stressful events; (3) employment of a housekeeper or caregiver with an employer-employee relationship with the care recipient. Drawing from previous studies (17, 18), we anticipated that a sample size of 300 would ensure reliable statistical outcomes for LPA. Consequently, a total of 380 questionnaires were distributed.

### Measures

#### Sociodemographic and clinical characteristics

Information on caregiver’s age, gender, marital status, education level, census register, work status, relationship with the patient, health status, economic burden, daily caregiving time, duration of caregiving, type of caregiving, medical insurance, and patient’s age was obtained through the questionnaire. Patient’s clinical information on clinical staging and course of disease was retrieved from patients’ medical records.

### Sense of coherence scale

The Chinese version of the Sense of Coherence Scale (SOC-13) (19) consists of 13 items and 3 dimensions, including meaningfulness (Items1, 9, 11, and13), comprehensibility (Items 2, 6, 7, 8, and 12), and manageability (Items3, 4, 5, and 10). Each item is rated on a 7-point Likert-type scale (from 1 = “strongly disagree” to 7 = “strongly agree”), with higher scores indicating higher levels of SOC. The total score of the scale ranges from 13 to 91 points, which can be divided into low level (13-63 points), medium level (64-79 points), and high level (80-91 points) based on the scoring range (20). The Cronbach’s alpha coefficient in this study was 0.845.

### Subjective well-being

The General Subjective Well-Being Schedule (GWB) was developed by Fazio et al (21). The Chinese version of the GWB (22) consists of 13 items and 6 dimensions, including energy level (Items1, 9, 14, and 17), satisfying interesting life (Items6, and11), emotional–behavioral control (Items3, 7, and13), relaxation and tension (Items2, 5, 8, and16), concerning about health (Items10, and 15), and depressed/cheerful mood (Items4, 12, and18). Among them, 1, 3, 6, 7, 9, 11, 13, 15, and 16 use reverse scoring. The scale ranges from 0 to 120 points, with higher scores indicating a better level of SWB. Specifically, scores from 0 to 24 points represent a low level of SWB, scores from 25 to 48 points indicate a relatively low level, scores from 49 to 72 points suggest a moderate level, scores from 73 to 96 points reflect a relatively high level, and scores from 97 to 120 points denote a high level of SWB. The Cronbach’s alpha coefficient in this study was 0.793.

### Procedure and quality control

Permission was granted by the administrators of the pertinent hospital departments, patients, and primary caregivers. The researcher personally distributed the questionnaire on-site, providing consistent instructions. The questionnaire was completed anonymously and autonomously by the patients’ primary caregivers. In cases where individuals had limited literacy skills or were unable to independently complete the questionnaire for other reasons, the researcher verbally presented the content and options in a neutral manner for them to select and complete the questionnaire with assistance. In order to ascertain the comprehensiveness and accuracy of the questionnaires, they were cross-checked on site for any omissions.

### Data analysis

Data analysis was conducted using SPSS 26.0 and Mplus 8.3. Conduct LPA on the items of SOC using Mplus 8.3 software. The fit indices mainly include (23, 24): (1) Akaike Information Criterion (AIC), Bayesian Information Criterion (BIC), and adjusted Bayesian Information Criterion (aBIC). The smaller the values of these three statistical indicators, the better the model fit. (2) Entropy is used to evaluate the accuracy of the model, with values ranging from 0 to 1. The closer the value is to 1, the more accurate the model fit. (3) The Lo-Mendell-Rubin Likelihood Ratio Test (LMRT) and Bootstrap Likelihood Ratio Test (BLRT) values are significant (*P*<0.05), indicating that a k-class model fits better than a (k-1) class model. (5) Models with a probability of at least 5% for each category are classified more reasonably. Multinomial regression analysis was conducted to explore demographic and clinical characteristic that influenced the latent classes of SOC. Finally, one-way analysis of variance (ANOVA) and *post hoc* tests (LSD test) with Bonferroni was performed to further determine the difference in GWB between different latent classes of SOC.

## Results

### Demographic and psychological characteristics

In total, 380 participants were recruited, and 360 questionnaires were returned (94.7%). We excluded 20 questionnaires because 8 people refused to complete the questionnaire or test, and 12 people did not conform to the criteria. Their mean age was56.8 ± 5.2, and 63.1% were male. Most were married 90.3%, were spouses of patients(73.6%), and lived in rural areas(63.1%). The comprehensive sociodemographic information, clinical characteristics of patients, and whether SOC differences among caregivers at different sociodemographic levels are significant are showed in **Table 1**. Moreover, common method bias was not observed in this study.

**Table 1:** Comparison of sociodemographic characteristics of caregivers in three profiles.

Variables	Profile 1 n (%)	Profile 2 n (%)	Profile 3 n (%)	χ2/F	*P*
Gender					
Male	17(6.7)	93(36.9)	142(56.3)	4.155	0.125
Female	14(13.0)	41(38.0)	53(49.1)		
Age (years)					
≤45	7(12.3)	19(33.3)	31(54.4)	11.652	**0.020**
45~65	15(7.0)	71(33.0)	129(60.0)		
≥65	9(10.2)	44(50.0)	35(39.8)		
Marital status					
Married	29(8.5)	125(36.3)	190(55.2)	3.546	0.170
Single	2(12.5)	9(56.3)	5(31.3)		
Education					
Primary school or below	15(15.8)	46(48.4)	34(35.8)	37.347	**<0.001**
Junior high school	7(4.5)	65(41.4)	85(54.1)		
High school	3(4.3)	14(20.3)	52(75.4)		
College	4(14.3)	5(17.9)	19(67.9)		
Bachelor's degree or above	2(18.2)	4(36.4)	5(45.5)		
Residential location					
Rural	6(5.2)	29(25.0)	81(69.8)	16.968	**<0.001**
Urban	25(10.2)	105(43.0)	114(46.7)		
Work status					
Employed	2(1.5)	35(26.3)	96(72.2)	42.091	**<0.001**
Unemployed	27(15.4)	80(45.7)	68(38.9)		
Retired	2(3.8)	19(36.5)	31(59.6)		
Relationship					
Parent	7(24.1)	13(44.8)	9(31.0)	11.351^a^	0.060
Spouse	20(7.5)	96(36.0)	151(56.6)		
Child	4(7.0)	23(40.4)	30(52.6)		
Other	0(0.0)	2(28.6)	5(71.4)		
Health status					
Poor	7(28.0)	11(44.0)	7(28.0)	40.169	**<0.001**
Average	17(10.3)	78(47.3)	70(42.4)		
Good	7(4.1)	45(26.5)	118(69.4)		
Financial burden					
Light	1(4.3)	3(13.0)	19(2.6)	54.245	**<0.001**
Medium	17(6.4)	85(32.0)	164(61.7)		
Heavy	13(18.3)	46(64.8)	12(16.9)		
Care timing					
<6h	4(5.7)	22(31.4)	44(62.9)	16.388	**0.012**
7~12h	12(6.5)	70(38.0)	102(55.4)		
13~18h	8(10.1)	30(38.0)	41(51.9)		
>19h	7(25.9)	12(44.4)	8(29.6)		
Care duration(months)					
<3	1(0.8)	41(31.3)	89(67.9)	29.255	**<0.001**
3~6	11(11.2)	33(33.7)	54(55.1)		
>6	19(14.5)	60(45.8)	52(39.7)		
Type of care					
Independent care	26(8.8)	111(37.6)	158(53.6)	0.261	0.878
Assisted care	5(7.7)	23(35.4)	37(56.9)		
Cancer stage					
I	2(4.3)	6(12.8)	39(83.0)	55.394	**<0.001**
II	3(1.9)	57(36.5)	96(61.5)		
III	13(12.1)	50(46.7)	44(41.1)		
IV	13(26.0)	21(42.0)	16(32.0)		
Duration of cancer					
1-3 (month)	3(2.4)	36(28.3)	88(69.3)	33.087	**<0.001**
3-6 (month)	10(9.1)	39(35.5)	61(55.5)		
6-12 (month)	5(9.6)	25(48.1)	22(42.3)		
1-5 (year)	9(20.5)	21(47.7)	14(31.8)		
>5 (year)	4(14.8)	13(48.1)	10(37.0)		

1) “^a^” represents Fisher exact probability, and the rest are χ^2^ values. 2) Bold values represent statistical significance.

### Classification of latent profile

This study fitted 3 potential profile models in total. Starting from the initial model 1 and successively increasing the number of model categories, a total of 1 to 5 category models were extracted, and the model fitting results are shown in **Table 2**. The values of AIC, BIC, and aBIC gradually decrease as the number of categories increases, and the entropy value is greater than 0.8. Both LMR and BLRT reached significance(*P*<0.05). However, Model 4 has category probabilities below 5%, suggesting that some categories may lack sufficient representation. Moreover, the Entropy value for Model 5 is the smallest. Therefore, considering all factors, it is determined that the 3-category model is the best fitting model. The average potential category probabilities for model 3 ranged from 0.919 to 0.956, confirming the high accuracy of the classification.

**Table 2:** Fitting indices of each model.

Model	AIC	BIC	aBIC	LMR	BLRT	Entropy	Profile prevalence
1	2654.947	2678.263	2659.228	–	–	–	–
2	2310.260	2349.121	2317.396	0.000	<0.001	0.826	38.0/61.9
**3**	**2189.338**	**2238.114**	**2199.328**	**0.0076**	**<0.001**	**0.869**	**7.9/37.3/54.7**
4	2163.540	2233.490	2176.385	0.0029	<0.001	0.876	3.1/7.1/53.5/36.3
5	2135.543	2221.037	2151.242	0.0024	<0.001	0.814	6.4/32.9/3.1/44.8/12.7

The bold values are the preferred model.

AIC, Akaike information criterion; BIC, Bayesian information; aBIC, sample size adjusted Bayesian information criterion; BLRT, Bootstrap likelihood ratio test; LMR, Lo–Mendell–Rubin; -, no such value.

**Figure 1:**
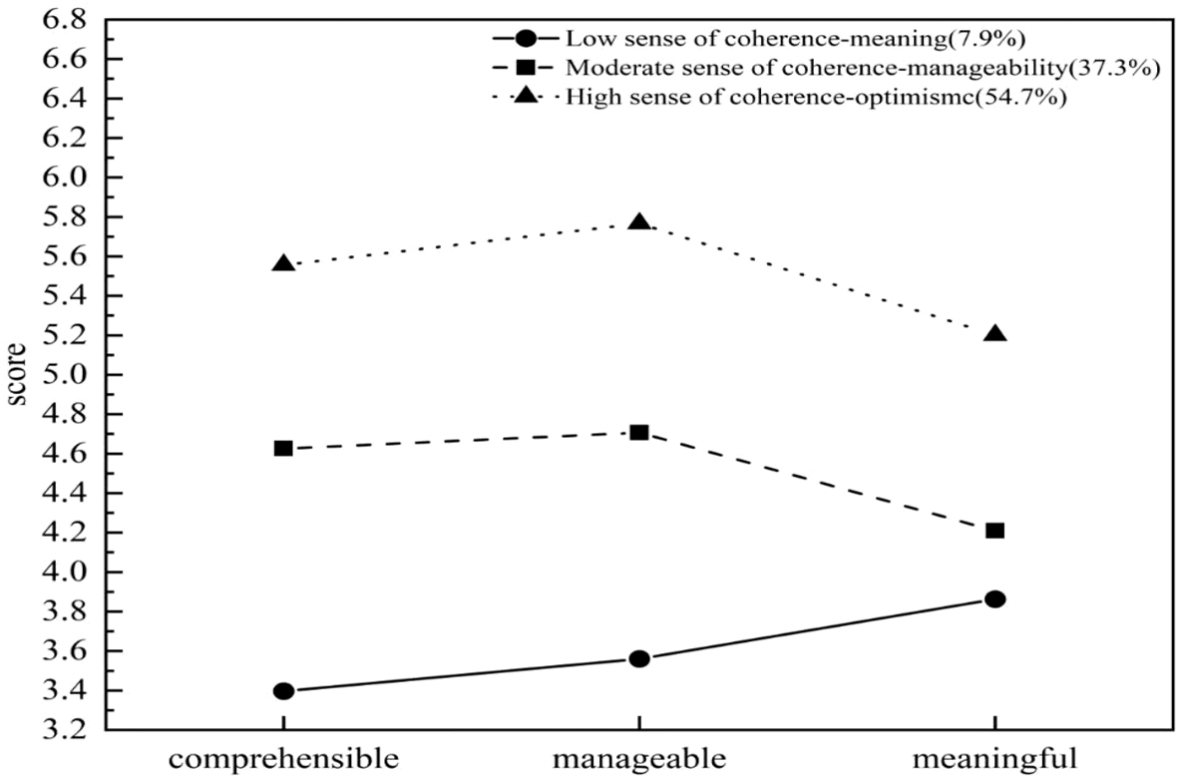
Latent profile of the SOC among caregivers of breast cancer patients.

Based on our research findings, we observed three distinct SOC characteristics among the caregivers (**Figure 1**). Profile 1 consists of 31 individuals (7.9%), with a total SOC-13 score of 46.71 ± 3.68, demonstrating low scores across all three dimensions, but they scored relatively high on the dimension of meaningfulness, hence it is designated as the “low sense of coherence-meaning group”. Results showed that these family caregivers held positive cognitions and valuable experiences towards the caregiving tasks and responsibilities they undertook, and were able to deeply experience that their efforts and dedication are meaningful and worthwhile.

Profile 2 consists of 134 individuals (37.3%), with a total SOC-13 score of 58.94 ± 3.86, demonstrating moderate level on various dimensions of SOC, but they scored relatively high on the dimension of manageability, hence it is designated as the “moderate sense of coherence-manageability group”. Results revealed that these family caregivers felt they were able to effectively control and responded to the situations and challenges related to their caregiving tasks or responsibilities, ensuring that the caregiving work proceeds in an orderly manner.

Profile 3 consists of 195 individuals (54.7%), with a total SOC-13 score of 71.84 ± 4.43, demonstrating scored highly on all dimensions of SOC, hence it is designated as the “high sense of coherence-optimism group”. Results indicated that this group possessed a high level of SOC, demonstrating strong adaptability and resilience when facing various situations and stressful events related to the cared-for individual. They maintained a positive and optimistic attitude throughout the caregiving process, effectively utilizing both internal and external resources to cope with potential difficulties and challenges, thereby providing more attentive and appropriate care.

### Associated factors of latent profile membership

Based on the three profiles, **Table 1** presents a comprehensive overview of the characteristics exhibited by caregivers of breast cancer patients. Our univariate analysis demonstrated statistically significant disparities in SOC classification among these caregivers, considering variables such as age, education, residence, work status, health status, financial burden, duration of cancer, Cancer stage, care duration, and care timing. Utilizing the three potential SOC categories as the dependent variables and Profile 3 as the benchmark, we conducted a multivariate regression analysis to identify the significant factors that influence the distribution of SOC among caregivers of breast cancer patients.

As shown in **Table 3**, the results revealed that care duration<3 month (OR=0.055, *P*=0.027), light financial burden(OR=0.037, *P*<0.05), medium financial burden (OR=0.201, *P*=0.016; OR=0.197, *P*<0.001), and clinical stage II (OR=0.046, *P*=0.003) had an increased likelihood of belonging to Profile 3. Further, residing in a rural area (OR=6.820, *P*=0.030), poor health status (OR=9.395, *P*=0.017), and age ≤ 44 (OR=17.344, *P*=0.023) had an increased likelihood of belonging to Profile 1. Additionally, average health status (OR=2.442, *P*=0.002) had a likelihood of belonging to Profile 2. These findings provide insights into the factors that influence the SOC distribution among caregivers of breast cancer patients, enabling a better understanding of their psychological well-being and the potential strategies for providing targeted support.

**Table 3:** Multinomial logistic regression of socio-demographic variables on subgroups of SOC.

Variables	Low sense of coherence-manageability group (profile 1)						Moderate sense of coherence-optimism group (profile 2)							
	β	S. E	Wald	P	OR	95%CI	β		S. E		Wald	P	OR	95%CI
Age (years)														
≤44	2.853	1.253	5.181	**0.023**	17.344	1.487-202.357	0.959		0.602		2.540	0.111	2.610	0.802-8.492
45-64	0.664	0.634	1.095	0.295	1.942	0.560-6.730	-0.299		0.380		0.620	0.431	0.742	0.352-1.561
≥65^a^	–	–	–	–	–	–	–		–		–	–	–	–
Education														
Primary school or below	-1.212	1.798	0.455	0.500	0.298	0.009-10.084	0.026		0.943		0.001	0.978	1.026	0.162-6.519
Junior high school	-3.116	1.808	2.971	0.085	0.044	0.001-1.533	-0.271		0.919		0.087	0.768	0.763	0.126-4.615
High school	-2.518	1.828	1.898	0.168	0.081	0.002-2.899	-1.167		0.937		1.551	0.213	0.311	0.050-1.954
college	-0.988	1.912	0.267	0.605	0.372	0.009-15.796	-1.674		1.004		2.778	0.096	0.188	0.026-1.342
Bachelor's degree or above^a^	–	–	–	–	–	–	–		–		–	–	–	–
Work status														
Employed	-2.841	1.537	3.416	0.065	0.058	0.003-1.188	-0.564		0.517		1.191	0.275	0.569	0.206-1.567
Unemployed	1.656	1.115	2.205	0.138	5.237	0.589-46.574	-0.195		0.491		0.158	0.691	0.823	0.314-2.152
Retired														
Rural	1.920	0.887	4.684	**0.030**	6.820	1.199-38.806	-0.061		0.392		0.024	0.877	0.941	0.436-2.030
Urban^a^	–	–	–	–	–	–	–		–		–	–	–	–
Care timing														
<6 h	-1.934	1.060	3.327	0.068	0.145	0.018-1.155	-0.877		0.644		1.857	0.173	0.416	0.118-1.469
7~12 h	-1.386	0.924	2.251	0.134	0.250	0.041-1.529	-0.591		0.599		0.974	0.324	0.554	0.171-1.791
13~18 h	-1.826	0.986	3.426	0.064	0.161	0.023-1.113	-1.105		0.648		2.906	0.088	0.331	0.093-1.180
>19h ^a^	–	–	–	–	–	–	–		–		–	–	–	–
Care duration (months)														
<3	-2.907	1.315	4.889	**0.027**	0.055	0.004-0.719	0.146	0.458		0.102		0.750	1.157	0.472-2.838
3~6	0.032	0.828	0.001	0.970	1.032	0.204-5.232	-0.336	0.478		0.496		0.481	0.714	0.280-1.822
>6 ^a^	–	–	–	–	–	–	–	–		–		–	–	–
Financial burden														
Light	-1.215	1.646	0.545	0.460	0.297	.012-7.476	-3.305	0.875		14.278		**<0.001**	0.037	0.007-0.204
Medium	-1.602	0.665	5.807	**0.016**	0.201	.055-0.742	-1.627	0.450		13.060		**<0.001**	0.197	0.081-0.475
Heavy ^a^	–	–	–	–	–	–	–	–		–		–	–	–
Health status														
Poor	2.240	0.936	5.727	**0.017**	9.395	1.500-58.845	1.053	0.605		3.024		0.082	2.865	0.875-9.386
Average	0.693	0.622	1.239	0.266	1.999	0.590-6.770	0.893	0.287		9.652		**0.002**	2.442	1.390-4.288
Good ^a^	–	–	–	–	–	–	–	–		–		–	–	–
Duration of cancer														
1-3 (month)	1.730	1.352	1.638	0.201	5.640	0.399-79.776	0.221	0.766		0.083		0.773	1.247	0.278-5.599
3-6 (month)	2.019	1.310	2.377	0.123	7.532	0.578-98.120	0.626	0.799		0.613		0.434	1.870	0.390-8.957
6-12 (month)	1.963	1.184	2.747	0.097	7.119	0.699-72.535	1.125	0.757		2.209		0.137	3.082	0.699-13.594
1-5 (year)	1.044	1.008	1.074	0.300	2.842	0.394-20.490	0.737	0.679		1.177		0.278	2.089	0.552-7.910
>5 (year) ^a^	–	–	–	–	–	–	–	–		–		–	–	–
Cancer stage														
I	-2.130	1.322	2.596	0.107	0.119	0.009-1.586	-1.164	0.726		2.574		0.109	0.312	0.075-1.295
II	-3.074	1.043	8.685	**0.003**	0.046	0.006-0.357	-0.039	0.545		0.005		0.942	0.961	0.331-2.796
III	-1.095	0.849	1.663	0.197	0.335	0.063-1.767	0.210	0.578		0.132		0.716	1.234	0.398-3.827
IV ^a^	–	–	–	–	–	–	–	–		–		–	–	–

1) ^a^ Reference category. 2) Bold values represent statistical significance.

### Relationships between the latent profiles of SOC and SWB

The caregivers of breast cancer patients scored in the General Well-being Schedule (GWB) as follows: energy level (14.63 ± 1.80), satisfying interesting life (5.97 ± 1.29), emotional-behavioral control (13.34 ± 1.60), relaxation and tension (17.20 ± 2.26), concerning about health (8.92 ± 2.39), and depressed/cheerful mood (19.80 ± 1.92). The differences in scores across various dimensions of SWB among caregivers of different categories were statistically significant (*P*<0.001). Further pairwise comparisons revealed that the scores of the Profile 3 in all dimensions of SWB were statistically significantly different from both Profile 1 and Profile 2(*P*<0.001). Similarly, pairwise comparisons between Profile 1 and Profile 2 also showed statistically significant differences (*P*<0.001). The comparison of SWB levels across different latent classes of SOC is summarized in **Table 4**.

**Table 4:** Comparison of dimensions and total scores of SWB among caregivers of breast cancer patients with different SOC profiles.

Variables	Profiles			F	*P*	LSD
	P1	P2	P3			
Total Score of Subjective Well-being	69.58 ± 6.00	76.20 ± 6.42	84.01 ± 5.90	113.09^a^	<0.001	P1<P2<P3
Concerning about health	7.84 ± 2.18	8.19 ± 2.24	9.58 ± 2.33	18.48^a^	<0.001	P1<P2<P3
Energy level	12.35 ± 1.60	14.22 ± 1.54	15.27 ± 1.62	52.08^a^	<0.001	P1<P2<P3
Emotional–behavioral control	12.00 ± 1.34	12.77 ± 1.61	13.94 ± 1.35	40.34^a^	<0.001	P1<P2<P3
Relaxation and tension	14.94 ± 2.56	16.46 ± 1.94	18.07 ± 1.98	46.71^a^	<0.001	P1<P2<P3
Depressed/cheerful mood	17.35 ± 1.82	19.10 ± 1.80	20.67 ± 1.42	79.06^a^	<0.001	P1<P2<P3
Satisfying interesting life	5.10 ± 0.98	5.46 ± 1.32	6.47 ± 1.09	87.00^a^	<0.001	P1<P2<P3

a The one-way ANOVA.

## Discussion

To our knowledge, this study was the first to employ LPA in exploring different profiles of SOC among caregivers of breast cancer patients, while simultaneously establishing the relationship between SOC profiles and GWB. The research revealed that LPA identified three profiles among caregivers: the “low sense of coherence-meaning group” (7.9%), the “moderate sense of coherence-manageability group” (37.3%), and the “high sense of coherence-optimism group” (54.7%). This suggested the existence of population heterogeneity in SOC among caregivers of breast cancer patients. Additionally, differences in demographic characteristics across different strata were observed, and various SOC profiles exhibited distinct associations with GWB. Generally, both caregivers in the Profile 2 and Profile 3 had significantly higher SWB compared with the Profile 1. This underscores the significance for healthcare professionals to consistently evaluate the caregivers of breast cancer patients, promptly and precisely identify those at high risk, and devise targeted intervention strategies that cater to the unique characteristics of each caregiver category.

It is noteworthy that the profile 1 accounted for 7.9% in this study. This group of caregivers exhibited a relatively low level of SOC in caring for patients, and the score of SOC-13 was consistent with the scoring criteria (20). Possible reasons included the physiological impact of surgical, chemotherapy, and radiotherapy treatments faced by breast cancer patients, which not only exacerbate the patients’ condition but also impose significant burden on caregivers, leading to reduced social interaction and even its discontinuation. This could result in economic pressure, emotional distress, and an imbalance in social roles (25), making caregivers subject to high caregiving stress. Caregiving stress could induce negative emotions such as anxiety and depression in caregivers, making it difficult for them to effectively mobilize available resources to cope with the negative impact of caregiving burden, resulting in poor psychological adjustment (26). However, they possessed a sense of self-affirmation and a sense of the meaning of life. Positive psychology suggested that individuals have the ability to experience positive psychological, cognitive, and emotional changes after experiencing traumatic events or situations (27). Research had shown that individuals with higher scores in meaning in life were able to reflect on their values, life purposes, and meanings in a timely manner when encountering any problems, effectively overcoming difficulties through continuous self-encouragement and reviewing past experiences (28). Therefore, enhancing the sense of meaning for this group of caregivers should be the primary focus of intervention. Healthcare professionals should actively acknowledge the value and significance of caregivers’ caregiving in daily nursing work, utilizing diversified strategies such as promoting friendship programs for caregivers of cancer patients, conducting individual and group counseling, and leveraging information and communication technologies to effectively integrate internal and external resources. This will help caregivers accept themselves, cultivate self-confidence, learn to evaluate themselves positively and objectively, ultimately enabling them to achieve both physical and mental well-being and provide better care for breast cancer patients.

The profile 2 accounted for 37.3% of the caregivers for breast cancer patients. While scoring moderately on all items related to SOC, they exhibited high scores on certain aspects of manageability, and the score of SOC-13 was lower compared to the established scoring criteria (20). This suggested that this group of caregivers possessed confidence in utilizing available resources to cope with stressful events and had the ability to perceive and control changes in their lives and environments. Therefore, enhancing their coping and control abilities should be the focus of intervention for this group. Bandura believed (29) that coping efficacy was an individual’s confidence in their ability to implement a series of actions or coping strategies to deal with external stress. Research has found (30) that under the same stressful conditions, individuals with high levels of coping efficacy were more confident in accepting the challenges brought by external pressure and maintaining a better SOC. According to the dual-factor model of caregiving experience (31), positive emotions mirrored the caregivers’ favorable assessments of caregiving tasks, which subsequently influenced their cognitive-behavioral reactions, specifically the coping mechanisms employed to manage stressful caregiving situations. Consequently, improving coping efficacy should be the primary focus of intervention for this group. Healthcare professionals can start by fostering positive feelings through self-affirmation and future outlook. On one hand, they can positively acknowledge the caregivers’ efforts and achievements, helping to enhance their sense of benefit and self-efficacy in caregiving. On the other hand, they can provide more positive examples of breast cancer patient outcomes, strengthening their confidence in the future, and ultimately enhancing their SOC.

In contrast to those in profile 1 and profile 2, 54.7% of the participants fell into profile 3, exhibiting the highest levels of SOC, and the score of SOC-13 was consistent with the scoring criteria (20). This group of caregivers was more inclined to perceive the positive aspects of caregiving, such as the health benefits it brought, which aligns with previous research findings (11). The salutogenic model also indicated (10)that SOC serves as a protective factor for mental health, enabling individuals to actively shift their attention away from negative cognitions and excel at adopting appropriate strategies to meet environmental demands, thereby investing in patient care with a more positive mindset.

The demographic characteristics exhibited notable variations across the three profiles of caregivers. Age, residence, and financial burden influence the profile of SOC. Firstly, caregivers aged ≤44 were more likely to be in the profile 1, which aligns with the findings of Mizuno et al (32). This was partly attributed to the fact that younger family caregivers tend to lack rich life experiences, possess a lower tolerance for life’s hardships, find it challenging to manage the pressures of daily life, and have limited expertise in patient care, ultimately leading to a higher preponderance of negative emotions. In contrast, older caregivers have more social experiences, are more likely to exhibit a calm mindset and stronger confidence in facing the stress of caregiving responsibilities, contributing to an enhanced SOC. Secondly, caregivers residing in rural areas were more prone to falling into the profile 1. This tendency stems from their limited material living conditions, weaker social support networks, and fewer resource access avenues compared to urban caregivers. Consequently, acquiring knowledge on caregiving techniques and mental health becomes challenging for them. These circumstances may undermine rural caregivers’ confidence when confronted with the responsibility of caring for patients, thereby diminishing their level of SOC. However, their profound affection for families and strong sense of responsibility instill in them a unique sense of value and meaning during caregiving, resulting in higher meaningfulness scores, aligning with previous research (33). Thirdly, compared to caregivers under heavy financial burdens, those under light and medium financial burdens were more likely to belong to the profile 3, which may be related to treatment period for breast cancer is long and costly. Caregivers who face lighter financial burdens often possess a stronger sense of control over their lives. They are able to mobilize a greater array of coping resources, which subsequently enhances their competency in executing caregiving tasks. This, in turn, improves their ability to manage stress. Conversely, families bearing heavier financial burdens are often burdened with more concerns and worries. They receive less social support and lack sufficient resources to tackle the challenges posed by caring for patients. These factors can erode their confidence in coping, ultimately making them more susceptible to having a low level of SOC.

In addition, caregivers of breast cancer patients with stage II were more likely to develop into profile 3. Cancer staging to some extent reflects the severity of the disease, prognosis, and survival time. The earlier the pathological staging, the better the treatment effect, and the higher the level of caregivers’ SOC (34). The reason for this lies perhaps in the fact that patients with lower cancer staging enjoy relatively superior treatment outcomes. This subsequently leads to a decrease in their dependence on caregivers, alleviating the burden of caregiving. In other words, it affords caregivers more time to relax, which in turn helps them rebuild confidence in the patient’s recovery and foster hope for future life. Furthermore, caregivers who provide care for less than 3 months were more likely to belong to the profile 3, which is consistent with existing research results (35). In the early stages of caregiving, tasks are relatively simple, and the stress is lower. Caregivers may feel a sense of novelty and responsibility, which can motivate them to be more involved and focused on caregiving, thereby enhancing their SOC. Additionally, caregivers with poor health status tend to belong to the profile 1, while those with average health status tend to belong to the profile 2.This may be because when caregivers experience poor physical health, such as physical exhaustion, sleep deprivation, fatigue, and negative emotions, it becomes challenging for them to provide satisfactory care. Despite their willingness to care, they may not be able to provide appropriate care at the right time, which can increase their care burden and impede the development of SOC (9).

The results of this study indicate that caregivers in the “high sense of coherence-optimism group” and the “moderate sense of coherence-manageable group” exhibited relatively high levels of SWB, while caregivers in the “low sense of coherence-meaningful group” scored at a moderate level of SWB. When comparing the SWB scores across various dimensions among the three caregiver groups, statistically significant differences were observed (*P* < 0.001). Furthermore, pairwise comparisons between the “high sense of coherence-optimism group” and both the “moderate sense of coherence-manageable group” and the “low sense of coherence-meaningful group” revealed statistically significant differences in SWB scores across all dimensions (*P* < 0.001). This suggests that caregivers with high total SWB scores and scores in its dimensions are more likely to belong to the “high sense of coherence-optimism group,” those with moderate scores are more likely to belong to the “moderate sense of coherence-manageable group,” and those with low scores are more likely to belong to the “low sense of coherence-meaningful group”, consistent with previous research findings (36). As an important component of positive psychology, caregivers with a high SOC tend to have a more positive view of stressful events, correctly understand and interpret the disease, are willing to put effort into overcoming caregiving difficulties, and focus more on the patient’s recovery rather than external opinions. Consequently, they are more resilient in facing difficulties during the caregiving process and less likely to perceive caregiving as a burden. Therefore, enhancing the level of SOC is crucial for improving the SWB of caregivers for breast cancer patients.

### Clinical implementation

The identification of caregivers’ SOC has revealed three distinct characteristics, “low sense of coherence-meaning group”、”moderate sense of coherence-manageability group” and “high sense of coherence-optimism group”. This study identified factors related to the SOC subgroups of caregivers, including age, residence, health status, financial pressure, caregiving duration, and breast cancer stage. Moreover, when considering the relationship between SOC and SWB, it is essential to evaluate SOC before developing intervention measures to improve SWB for caregivers. This implies that healthcare professionals should take into account not only the total score of the scale but also the specific manifestations when assessing caregivers’ SOC. To maximize the effectiveness of the intervention, tailored intervention measures should be designed and implemented based on the characteristics of individual SOC categories.

### Limitations

This study has some limitations. Firstly, it was the first time that LPA had been used to explore the SOC among caregivers of breast cancer patients with different characteristics. The sample size for some caregiver categories was relatively small, and the study was limited to one tertiary hospital. Therefore, the persuasiveness and generalizability of the latent category analysis results were still insufficient. Secondly, a cross-sectional survey could not establish a causal relationship between influencing factors and latent profiles, nor can it infer the causal relationship between SOC and SWB. Thirdly, the majority of study participants being male spouses limits the generalization of the research findings. Future studies could expand the sample size, consider a balanced gender ratio and conduct multicenter longitudinal research to explore the dynamic trajectories of caregivers and the long-term predictive effects of SOC on SWB, thereby facilitating targeted clinical interventions for SOC.

## Conclusions

Our study has identified three distinct latent categories of SOC among caregivers of breast cancer patients using LPA, thereby enhancing our understanding of the heterogeneity within this population. It is imperative for healthcare providers to prioritize attention towards caregivers who are young, reside in rural areas, are in suboptimal physical condition, and are burdened by significant economic pressures. To alleviate their caregiving burden, enhance their SOC, and ultimately improve their SWB experience, targeted and effective intervention measures must be implemented.

## Data Availability

The raw data supporting the conclusions of this article will be made available by the authors, without undue reservation.
